# Multiple sclerosis patients have reduced resting and increased activated CD4^+^CD25^+^FOXP3^+^T regulatory cells

**DOI:** 10.1038/s41598-021-88448-5

**Published:** 2021-05-18

**Authors:** Nirupama D. Verma, Andrew D. Lam, Christopher Chiu, Giang T. Tran, Bruce M. Hall, Suzanne J. Hodgkinson

**Affiliations:** 1 Ingham Institute for Applied Medical Research, Liverpool, NSW Australia; 2 Immune Tolerance Laboratory, Department of Medicine, University of New South Wales, Ingham Institute for Applied Medical Research, 1 Campbell Street, Liverpool, NSW 2170 Australia; 3 Multiple Sclerosis Clinic, Department of Neurology, Liverpool Hospital, Locked Bag 7103, Liverpool, NSW 1871 Australia

**Keywords:** Autoimmunity, Multiple sclerosis, Lymphocyte activation

## Abstract

Resting and activated subpopulations of CD4^+^CD25^+^CD127^lo^T regulatory cells (Treg) and CD4^+^CD25^+^CD127^+^ effector T cells in MS patients and in healthy individuals were compared. Peripheral blood mononuclear cells isolated using Ficoll Hypaque were stained with monoclonal antibodies and analysed by flow cytometer. CD45RA and Foxp3 expression within CD4^+^ cells and in CD4^+^CD25^+^CD127^lo^T cells identified Population I; CD45RA^+^Foxp3^+^, Population II; CD45RA^−^Foxp3^hi^ and Population III; CD45RA^−^Foxp3^+^ cells. Effector CD4^+^CD127^+^ T cells were subdivided into Population IV; memory /effector CD45RA^−^ CD25^−^Foxp3^−^ and Population V; effector naïve CD45RA^+^CD25^−^Foxp3^−^CCR7^+^ and terminally differentiated RA^+^ (TEMRA) effector memory cells. Chemokine receptor staining identified CXCR3^+^Th1-like Treg, CCR6^+^Th17-like Treg and CCR7^+^ resting Treg. Resting Treg (Population I) were reduced in MS patients, both in untreated and treated MS compared to healthy donors. Activated/memory Treg (Population II) were significantly increased in MS patients compared to healthy donors. Activated effector CD4^+^ (Population IV) were increased and the naïve/ TEMRA CD4^+^ (Population V) were decreased in MS compared to HD. Expression of CCR7 was mainly in Population I, whereas expression of CCR6 and CXCR3 was greatest in Populations II and intermediate in Population III. In MS, CCR6^+^Treg were lower in Population III. This study found MS is associated with significant shifts in CD4^+^T cells subpopulations. MS patients had lower resting CD4^+^CD25^+^CD45RA^+^CCR7^+^ Treg than healthy donors while activated CD4^+^CD25^hi^CD45RA^−^Foxp3^hi^Treg were increased in MS patients even before treatment. Some MS patients had reduced CCR6^+^Th17-like Treg, which may contribute to the activity of MS.

## Introduction

The cause of Multiple Sclerosis (MS) is not fully understood. Auto-reactive inflammatory cells including effector T cells (Th1, Th17, CD8^+^ Cytotoxic T cells), activated B cells, and plasma cells producing autoantibodies infiltrate the central nervous system (CNS). This leads to patchy myelin and axonal damage in the CNS. Susceptibility to MS is strongly linked to Class II MHC haplotypes^1,2^, which present antigens to CD4^+^T cells. Autoimmunity can occur due to failure of Treg that express Foxp3 and have low expression of CD127^3^. Many genes associated with MS relate to CD4^+^CD25^+^Foxp3^+^ T regulatory cells (Treg), including *CD25, Ctla4, CD127, Il10*^1,2^. Treg have the potential to limit inflammation in MS.

There are conflicting reports on Treg in blood of MS patients. A meta-analysis of eight papers reported CD4^+^CD25^+^Foxp3^+^Treg in blood were not reduced in MS^4^. Other studies found Treg function is not affected in MS^5–8^.

There are reports of defects in/or deficiency of Treg in blood^7–20^, brain and CSF^13^ of MS patients. Treg in MS patients have reduced message and protein expression of Foxp3^10,12,21–23^, are functionally impaired^9–12,14,23,24^ with poor suppression of T cell responses to myelin basic protein^25,26^.

In contrast, higher Treg compared to healthy donors (HD)^20^ are described in secondary progressive MS patients. Higher frequency of Treg have been observed in cerebrospinal fluid (CSF) compared to blood in MS^10,13,24^, whereas others reported no difference in CSF and blood^25^.

Human Treg are best defined as CD4^+^CD25^+^CD127^lo^Foxp3^+^ but use of Treg makers is not consistent. In 29 studies we identified of CD4^+^CD25^+^Treg in blood of MS patients^5–12,14–17,19,20,22–25,27–37^, only 20 stained for Foxp3^6–8,10–12,14,15,17,19,22–24,28–32,34,36^ and 14 for CD127^6,11,14,16,19,20,23,30,31,33–37^. CD4^+^CD25^+^CD127^lo^ Foxp3^+^T cells are a heterogeneous population that can include Treg but also includes activated effector cells that are transiently induced to express CD25 and Foxp3 but this Foxp3 expression is unstable^38–41^. Thus, many studies on CD4^+^CD25^+^Foxp3^+^T cells include a proportion of activated T cells that do not suppress.

A variety of markers have been used to subdivide Treg, including CD31 expression as a marker of Treg of recent thymic origin^14,29^. Attempts to identify activated Treg include 11 studies of CD45RA/RO expression^11,14,22,24,27,29,31,33–35,37^, eight of CD39 expression^7,16,22,28,30,32,36,37^, four of HLA-DR expression^27,33,35,37^, one of CD161 expression^34^, and one of PD-1 expression^22^.

Relevant to this study, CD4^+^CD25^+^CD127^lo^ Foxp3^+^T cells can be divided into subpopulations by their expression of CD45RA, an isoform of CD45. Expression of CD45RA is reduced as Treg are activated^42,43^ and other isoforms of CD45 including CD45RB, CD45RC and CD45RO^44^ are expressed. Treg activation also increases expression of CD25 and Foxp3.

**Figure 1:**
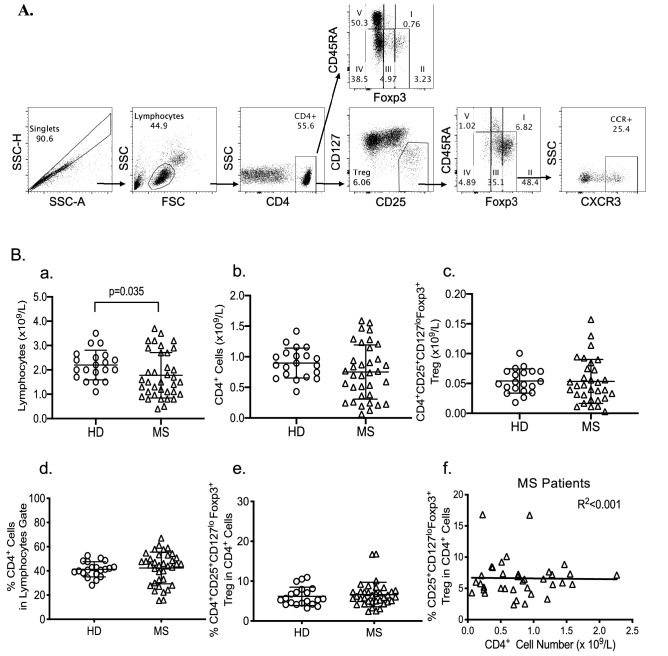
(**A**) Gating Strategy for analysis of CD4^+^ and Treg subpopulations: PBMC isolated from a MS patient were stained for multicolour flow cytometry. Cells were first gated on SSC-A vs. SSC-H to exclude doublets, then lymphocytes were gated based on forward scatter (FSC-A) and side-scatter (SSC-A). CD4^+^ cells within the lymphocyte population were gated. Staining of CD25 vs. CD127 identified CD4^+^CD25^+^CD127^lo^Treg within CD4 gate. Subpopulations were then identified based on Foxp3 vs. CD45RA expression either from CD4^+^ gate or from CD4^+^CD25^+^CD127^lo^Treg gate. Within CD4^+^ cells five different populations were studied as defined by Miyara et al.^43^. Treg are thought to be located within populations I, II and III, with activated Th-like Treg thought to lie within population II. Populations IV and V are Foxp3^−^ and CD25^−^ and are respectively defined as activated/memory (CD45RA^−^) and effector CD4^+^ cells (CD45RA^+^). Populations I, II and III were then analysed for chemokine receptor expression (CXCR3, CCR6 or CCR7) to examine frequencies of specific Th-like Treg subsets, showing CXCR3 Th1-like Treg as an example. (**B**): Comparison of percentage of lymphocyte subsets in HD and MS patients. PBMC from MS patients (n = 36) were compared to PBMC from HD (n = 20) for lymphocyte count (a), CD4^+^cell count (b) and their proportion within lymphocytes (d), and CD4^+^CD25^+^CD127^lo^Foxp3^+^ Treg count (c) and their proportion within CD4^+^cells (e). Regression analysis for Treg proportion within CD4^+^ cells vs. CD4^+^ cell numbers did not change with change in CD4^+^ cell numbers (f).

CD4^+^T cells can be divided into five populations using CD45RA and either Foxp3 or CD25 expression^37,43^ (Fig. 1A). Population I mainly contains thymus derived resting Treg that express CD45RA, CD25 and Foxp3 and is large at birth but decreases with age^42^. Population II identifies activated Treg that are CD45RA^−^ with high expression of CD25 and Foxp3. These cells are activated/memory Treg. Population III includes cells that are CD45RA^−^ and have low to intermediate expression of Foxp3 and CD25. Population III includes effector CD4^+^T cells with transient expression of CD25 and Foxp3, that are activated to secrete inflammatory cytokines and do not suppress^43^. Population IV includes activated effector CD4^+^T cells that do not express CD45RA, CD25 or Foxp3. Population V identifies CD4^+^ cells with high CD45RA expression and no CD25 and Foxp3.

Activation of CD4^+^ T cells would expand Population IV while activation of Treg would increase Population II and III and may deplete Population I.

The five subpopulations identified by CD45RA, Foxp3 or CD25 expression can be subdivided by staining for other cell surface molecules. In this study, we examined key chemokine receptor (CCR) expression, which changes with activation of T cells. In Population I, expression of CCR7 identifies resting Treg that are recent thymic immigrants, whereas cells in Population I lacking CCR7 expression are effector memory (EM) Treg^45,46^.Terminally differentiated effector memory cells that re express CD45RA, but not CCR7, are known as TemRA cells^47^ and are found in Population V together with naïve CD4^+^ cells that are CD45RA^+^CCR7^+^.

Depending on the stimulating antigen, naïve CD4^+^ T helper lineage cells can be activated by cytokines to Th1, Th2, Th17 and Tfh effector cells. Th1 and Th17 cells mediate autoimmunity including MS^48^. Chemokine receptors expressed by activated T effector cells promote their migration to inflamed tissue. Th1 cells express CXCR3^49^ and Th17 cells express CCR6^50^.

Similar to T effector cell activation, CD4^+^CD25^+^Foxp3^+^Treg can be activated by antigen and cytokines of the ongoing effector response^51,52^ to develop into Th1-like, Th2-like, Th17-like or Tfh-like Treg^53–56^. Activated Treg express the same chemokine receptors as their associated activated T cells^53^. Treg expressing CXCR3 or CCR6 respectively migrate to sites of Th1 or Th17 inflammation in tissue^57,58^.

We examined peripheral blood lymphocytes, either CD4^+^ cells to identify Populations I-V or CD4^+^CD25^+^Foxp3^+^CD127^lo^ Treg to identify Populations I–III. We also studied CCR expression within each of subpopulations of CD4^+^ cells and Treg. We compared these blood lymphocytes of healthy donors (HD) to MS patients to examine if this method of monitoring may reveal changes that could provide insights into the immune pathogenesis of MS and the effects of therapy. Three groups of MS patients were recruited; (i) patients newly diagnosed with MS that had received no treatment, (ii) patients whose MS had been inactive and who had received no immunomodulating therapy for three months and (iii) patients with clinical activity of MS that were on immunomodulating therapy.

MS patients’ blood had lower Population I (CD45RA^+^Foxp3^+^) and increased Population II (CD45RA^−^Foxp3^hi^). MS patients had less CCR6^+^ cells in Population III and some MS patients had lower numbers of CCR6^+^ cells in Population II. MS patients’ blood also had less CD4^+^ Population V including CD45RA^+^CCR7^+^ effector cells and TemRA cells and more activated/memory effector CD4^+^T cells (Population IV). These changes were evident in all three clinical categories and appear to be due to the disease of MS, not other factors.

## Human ethics and subjects

This study was approved by the Human Ethics Committee of the South Western Sydney Local Health District at Liverpool Hospital, Liverpool, NSW Australia, and complied with the Declaration of Helsinki-Ethical Principles for Medical Research involving Human Subjects. All subjects voluntarily gave informed written consent.

HD were recruited by an internal email invitation within the hospital. All volunteers were screened to confirm the absence of diseases such as autoimmune conditions, HIV, other infections and anaemia. 20 healthy donors (HD) and 36 MS patients were recruited, and their characteristics are detailed in Table 1.

**Table 1:** Characteristics of healthy donors (HD) and multiple sclerosis (MS) patients.

	Total HD	MS				
		Total MS	Active^3^	Non-active^4^	Recent treatment	No recent treatment^5^
N	20	36	22*	12*	15*	20*
Age-mean (range) years	41.7 (21–69)	42.5 (21–64)	40 (21–61)	45.6 (26–64)	42.2 (21–61)	42.1 (22–64)
Male	6	9	6	2	3	6
Female	14	27	16	10	12	14
Disease Duration^1^ years	N/A	8.9 (0.1–25.2)	7.9 (0.1–25.2)	11.5 (4.8–24.9)	9.2 (0.1–25.3)	8.3 (0.1–19.4)
EDSS^2^	N/A	3.71 (0–8.5)	3.24 (0–6)	4.2 (1–6.5)	3.04 (0–6)	4.21 (1–6.5)

^1^Disease duration calculated from very first onset of symptoms to time of study.

^2^EDSS is expanded disability status scale.

^3^Active MS defined as relapse or clinical progression of MS in 3 months before study.

^4^Non active MS, no progression or relapse in last 6 months.

^5^No recent treatment—no immunomodulatory therapy received in 3 months before study.

*Some clinical data not available for this post-hoc analysis.

All MS patients had a clinical diagnosis of MS made according to the McDonald Criteria 2017^59^. Total 36 MS patients were recruited including recently diagnosed MS patients and patients who had received prior treatment with immunomodulatory drugs. Patients were assigned to three groups: (i) a treatment naïve group of 12 patients who had never received treatment with immune modulating therapies, including interferon β (Treatment Naïve), (ii) 8 MS patients who were clinically stable and had not received immune modulating therapy in the last three months, (iii) 15 MS patients who had received immune modulating therapy in the last three months (on therapy). For some analysis, Group I and II were combined as one group of patients not currently on immune modulating therapy and clinically stable (off therapy, n = 20). MS patients were defined to have active disease if they had a relapse or deterioration of symptoms within the last 3 months of sampling.

Immune modulating therapy included 15 patients given interferon-β, all had stopped this therapy 18 months to 10 years prior. Four patients had received glatiramer acetate (Copaxone, Teva Pharma Australia), which had been stopped 3-6 years ago. Eight patients had received natalizumab (Tysabri, Biogen Australia): two stopped 4 weeks prior sampling, the others 8 months to 6 years prior. 14 patients had been treated with fingolimod (Gilenya, Novartis Australia), nine had stopped this therapy in the previous 3 months, the other five stopped 2-4 years prior sampling. Five patients had received dimethyl fumarate (Tecfidera, Biogen Idec Australia), two were currently on the drug and three stopped over a year ago. Five patients had been treated with Rituximab (Mabthera, Roche, Australia) 7–12 months prior sampling. Four patients were treated with alemtuzumab (Lemtrada, Sanofi Aventis Australia), two a year prior and the other two 7–8 years ago. Two patients had been treated with cladribine (Mavenclad, Merck Australia), one remained on the drug, and the other had it a year prior sampling. One patient received IvIg (CSL, Melbourne Australia) 4 months prior. One patient received teriflunomide (Aubagio, Sanofi Aventis, Australia) 3 years prior. One patient had received methotrexate and another an unsuccessful stem cell transplant in Mexico, both several years prior. No patient had received anti-CD25 monoclonal antibody therapy.

## Materials and methods

### Cell isolation

Peripheral blood lymphocyte counts (PBL) were assessed by a clinical haematological laboratory. Peripheral blood mononuclear cells (PBMC) were isolated from 30ml of fresh whole blood using Ficoll-Hypaque density gradient centrifugation (Ficoll-Paque^TM^ PLUS, GE Healthcare Bio-Sciences AB).

### Immunostaining

Monoclonal antibodies used were to CD4 (RPA-T4, APC-Cy7), CD127 (HIL-7R-M21, PE- Cy7), CD25 (M-A251, PE-Cy7), CXCR3 (IC6, PE), CCR6 (11A, PE), CCR7 (150503, PE); all purchased from BD Biosciences (North Ryde, NSW, Australia), CD45RA (HI100, APC) (Biolegend, San Diego, CA) and Foxp3 (PCH101, AF488) (eBioscience, San Diego, CA).

Fresh PBMC (1 × 10^6^) were stained for chemokine receptors for 15 minutes at room temperature in dark before staining for CD4, CD25, CD127 and CD45RA for 30 minutes on ice. Cells were washed, fixed and permeabilized according to the manufacturer’s protocol (eBioscience) then stained for intracellular Foxp3.

Phenotypic analysis was performed on stained PBMC using a FACSCanto II flow cytometer (BD Biosciences) and FACS DIVA 8.0 software. Data was analysed following the gating strategy shown in Fig. 1A using FloJo v10 software (Tree Star, Ashland, OR). Lymphocyte populations were gated after exclusion of doublets based on SSC-A vs. SSC-H.

CD4^+^ cells were divided into five populations based on CD45RA and Foxp3 expression as described by Miyara et al.^43^. The percentage of each population was also calculated within CD4^+^CD25^+^CD127^lo^Treg, which mainly has cells in Population I, Population II and Population III (Fig. 1A).

Each population was further examined for the Th-like Treg phenotype using expression of chemokine receptors; CXCR3 for Th1-like Treg, CCR6 for Th17-like Treg and CCR7 for circulating naïve Treg. The gating for chemokine receptor expressing cells was based on a Flourescence Minus One (FMO) control.

### Statistical analysis

Statistical analysis was performed using Graphpad Prism 8.0.2 and IBM SPSS Statistics 25. For comparison of unpaired data, the Mann–Whitney U test was used. The Kruskal–Wallis Test was used in cases where multiple independent groups were assessed. Linear regression analysis was performed to compare the effect of age and disease duration with Treg parameters. Results were expressed as mean ± SEM unless otherwise specified and significance was *p* < 0.05.

## Results

### Comparison of peripheral lymphocyte counts (PBL) in HD and MS patients

The demographic characteristics of HD and MS patients are summarised in Table 1. HD had higher PBL counts than MS patients, 2.19 ± 0.60 (mean ± SEM) vs. 1.77 ± 0.15  × 10^9^/L (*p* = 0.035) (Fig. 1Ba). MS patients with no previous therapy had PBL counts of 2.45 ± 0.25  × 10^9^/L that was not significantly different to HD. Compared to HD, PBL counts were lower in MS patients whose disease was inactive (*p* = 0.004) or had treatment in last three months (*p* = 0.012), but not in those not treated in last three months (*p* = 0.054). Regression analysis of PBL counts showed no significant effect of age, disease duration or time since last flare of MS (R^2^ = 0.053, *p* = 0.316).

### Comparison of peripheral CD4^+^T cell counts in HD and MS patients

CD4^+^ cell counts for HD and MS were not significantly different (Fig. 1Bb). Patients with active MS had lower CD4^+^ counts (0.602 ± 0.074 × 10^9^/L) whereas MS patients with no previous therapy had CD4^+^ counts (1.065 ± 0.154 × 10^9^/L) similar to HD (0.990 ± 0.088 × 10^9^/L). There was no difference in the proportion of CD4^+^ cells in PBL between MS patients and HD (42.14% ± 13.14% vs. 41.26% ± 6.19%, *p* = 0.339) though the variation within MS patients was greater than in HD (Fig. 1Bd).

Patients with active MS had higher numbers and proportions of CD4^+^ cells compared to those with inactive disease (*p* = 0.02). CD4^+^ cell counts or proportion of CD4^+^ cells (*p* = 0.184) were not different in MS patients that received therapy in last three months compared to MS patients who received no therapy in the last three months. Regression analysis found neither the number nor the proportion of CD4^+^ cells in MS patients correlated with age, disease duration or time since last flare of MS (R^2^ = 0.109, *p* = 0.087).

### Comparison of peripheral CD4^+^CD25^+^CD127^lo^T cells in HD and MS patients

The number of CD4^+^CD25^+^CD127^lo^Foxp3^+^cells in MS was not significantly different to HD (*p* = 0.472, Fig. 1Bc). However, the spread of counts was wider in MS patients as a proportion of MS patients had either low or higher absolute numbers. There was no difference between HD and MS in the proportion within CD4^+^ cells of CD4^+^CD25^+^CD127^lo^ (7.20 ± 0.546% vs. 7.48 ± 0.502%, *p* = 0.815) or of CD4^+^CD25^+^CD127^lo^Foxp3^+^ (6.19 ± 0.516 vs. 6.62 ± 0.516%, *p* = 0.573, Fig. 1Be).

Regression analysis of CD4^+^ cell numbers with proportion of Treg in CD4^+^ cells in MS patients showed no correlation (R^2^ = 0.001) (Fig. 1Bf). This demonstrated that the ratio of Treg as a proportion of CD4^+^ cells did not relate to total CD4^+^ cell number. Thus, all results were expressed either as proportions of either CD4^+^ or of CD4^+^CD25^+^CD127^lo^Treg.

### Comparison of the three Treg populations based on CD45RA and Foxp3 expression in HD and MS patients

**Figure 2:**
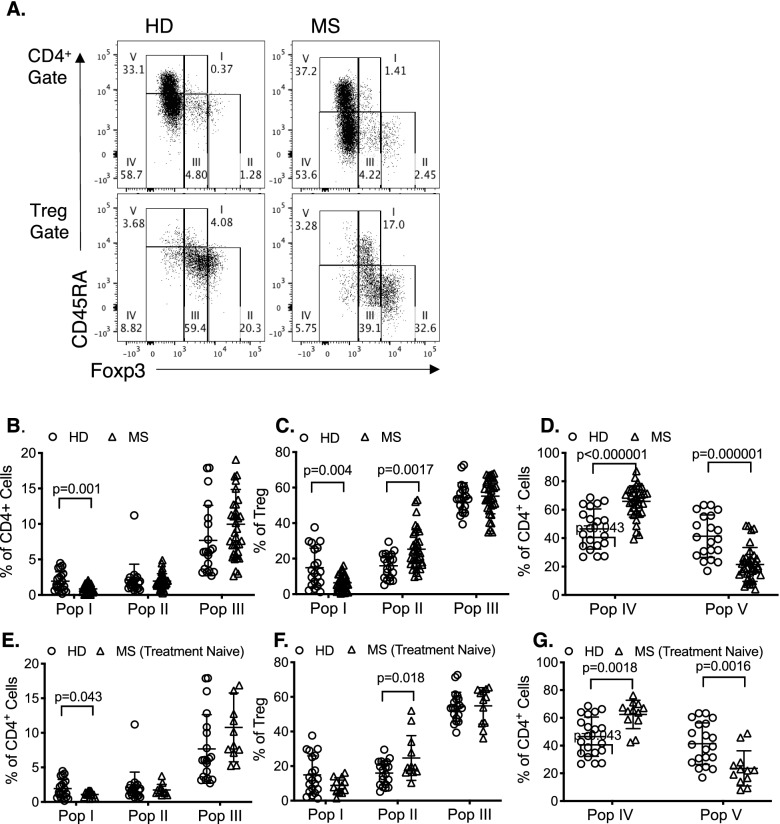
Comparison of Treg subpopulations in HD and MS patients. Subpopulations were gated based on CD45RA and Foxp3 either within CD4^+^ cells (Population I-V) or CD4^+^CD25^+^CD127^lo^Treg (population I–III). Values were expressed as the percentage of cells within each population. (**A**) Representative FACS profile of PBMC from HD (Left Column) and an MS patients (Right Column) gated on CD4^+^ (Top Row) and CD4^+^CD25^+^CD127^lo^Treg (Bottom Row) showing increased frequency of Foxp3^hi^CD45RA^−^ cells in Population II in MS patients compared to HD; 2.45% vs. 1.28% when gated on CD4^+^, and 32.6% vs. 20.3% when gated on CD4^+^CD25^+^CD127^lo^ Treg. (**B**–**D)** shows comparison of all MS patients (n = 36) with HD (n = 20). (**B**) CD4^+^ cells had lower proportion of Population I (*p* = 0.001) in MS (n = 36) than HD (n = 20). (**C**) As proportion of CD4^+^CD25^+^CD127^lo^Treg, significant difference was noted both in population I and II, Population I was significantly lower (*p* = 0.004) and Population II was significantly greater (*p* = 0.0017) in MS (n = 36) than HD (n = 20). (**D**) Proportion of Population IV (activated/memory effectors) in CD4^+^ was greater in MS (n = 36) compared to HD (n = 20) (*p* < 0.000001). Proportion of Population V (Foxp3^−^CD45RA^+^) within CD4^+^ cells was significantly lesser in MS than in HD (*p* < 0.000001). (**E–G)** shows comparison of subpopulations in treatment naïve MS patients (n = 12) to HD (n = 20) using similar analysis as in B–D. (**E**) Treatment naïve MS patients had significantly lower proportion of cells in Population I in CD4^+^ gated cells than HD (*p* = 0.043). There was no significant difference between Population II or Population III. (**F**) In CD4^+^CD25^+^CD127^lo^Treg gate Population II was significantly greater in untreated MS than HD (*p* = 0.018) but there was no difference in population I or III although Population I was low. (**G**) In CD4^+^ gated cells, MS patients had more Population IV (*p* = 0.0018) and less Population V (*p* = 0.0016) than HD.

We next examined if there were changes in the subpopulations of CD4^+^ cells and CD4^+^CD25^+^CD127^lo^ Treg as described by Miyara et al^43^ for CD4^+^ cells identifying resting and activated Treg by staining for CD45RA and Foxp3/CD25. A representative plot of five subpopulations within CD4^+^ cells and CD4^+^CD25^+^CD127^lo^Treg population is shown as Fig. 2A. To identify if changes demonstrated in studies of all MS patients (Fig. 2B–D), were a marker of immune changes intrinsic to MS or a consequence of immune modifying therapy, we compared CD4^+^ and CD4^+^CD25^+^CD127^lo^ Treg from treatment naïve MS patients (n = 12) and HD (Fig. 2E–G).

CD45RA^+^Foxp3^+^T cell (Population I) proportion within CD4^+^ cells was significantly smaller in MS than HD when comparing either all MS patients (*p* = 0.001) (Fig. 2B) or treatment naïve MS patients (*p* = 0.043) (Fig. 2E).

Within CD4^+^CD25^+^CD127^lo^Treg, Population I was also significantly smaller in MS than HD (*p* = 0.004) (Fig. 2C). Population I was significantly lower in MS patients who were off treatment in last three months compared to HD (*p* = 0.008), but not for those who were on treatment in the last three months (data not shown).

CD4^+^CD25^+^CD45RA^−^Foxp3^hi^ cells (Population II), the most suppressive of the three Populations^43^, was significantly increased in CD4^+^CD25^+^CD127^lo^Treg in all MS patients (n = 36, *p* = 0.0017) (Fig. 2C) and treatment naïve MS patients (n=12, *p* < 0.018) (Fig. 2F) compared to HD. However, there was no difference between MS and HD (*p* = 0.532) in Population II of CD4^+^T cells either comparing all MS (Fig. 2B) or treatment naïve patients to HD (Fig. 2E).

There was no significant difference of Population III in either CD4^+^ cells (Fig. 2B,E) or CD4^+^CD25^+^CD127^lo^Treg (Fig. 2C,F), when comparing all MS or treatment naïve MS to HD. Population III includes activated effector CD4^+^ that are activated and transiently express Foxp3 and CD25^41,60^.

### Changes in effector CD4^+^T cells in MS compared to HD

Comparing all MS patients with HD, Population V containing naïve CD4^+^CD45RA^+^Foxp3^−^ and TemRA cells was significantly lower in MS patients (21.48 ± 11.90%) than in HD (41.34 ± 14.07%) (*p* = 0.000001) (Fig. 2D). The TemRA CD4^+^CD245RA^+^CCR7^−^ cells in Population V, were 12.25 ± 23.30% in MS and 5.16 ± 8.46 in HD. Population IV, CD4^+^CD45RA^−^Foxp3^−^ activated effector T cells, was increased in all MS (65.85 ± 11.02%) compared to HD (46.42 ± 14.45%) (*p* < 0.000001) (Fig. 2D).

These differences were also observed when treatment naïve MS patients (n=12) were compared to HD (Fig. 2G). Population IV was significantly increased in MS (*p* = 0.0018) and Population V was significantly decreased in MS patients (*p* = 0.0016), (Fig. 2G).

**Figure 3:**
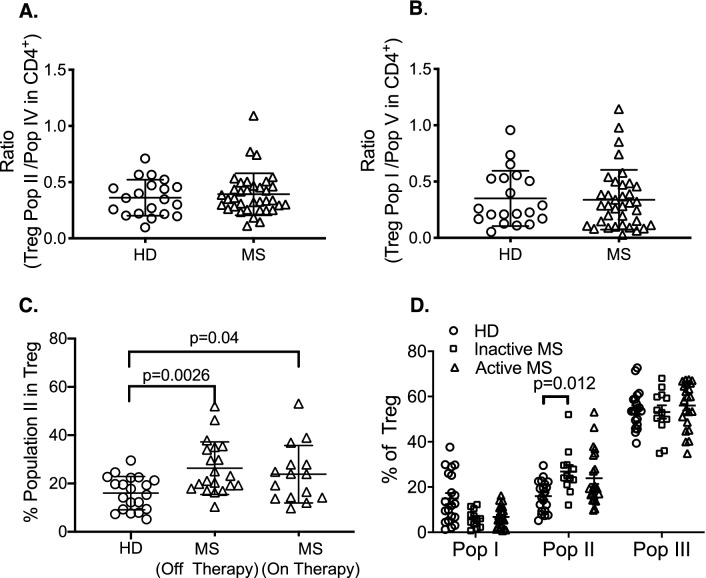
Relationship of Treg to effector cell subpopulations and treatment in HD and MS patients. (**A**) Ratio of Treg Population II to activated/memory effector cells in population IV was examined. No significant difference was observed in MS (n = 36) compared to HD (n = 20). (**B**) Ratio of Treg Population I to effector cells in Population V found no significant difference between HD (n = 20) and MS patients (n = 36). (**C**) Comparison of Population II in Treg in HD (n = 20) and MS patients that either had no recent immunomodulatory therapy (n = 20) or received immunomodulatory therapy in last three months (n = 15). Significant differences were found between HD and MS patients who were off therapy (*p* = 0.0026) or were on immune modulating therapy for MS in the last three months of sampling (*p* = 0.04). (**D**) Comparison of Treg subpopulations in HD (n = 20), to patients with active MS (n = 22) and those who did not have active MS (n = 12). MS was classified as active if patients reported a clinical relapse or deterioration of symptoms within the last 3 months. Significant difference was observed in Treg Population II in MS patients with inactive disease compared to HD(*p* = 0.012). Patients with active MS, did not have an increase in Population II, albeit five of 20 patients had increased number of Population II above mean value for HD.

The ratio of Population I to Population V was not different between HD and MS (*p* = 0.867) (Fig. 3B). The ratio of activated Treg Population II to activated effector Population IV also showed no difference between HD and MS (*p* = 0.188) (Fig. 3A).

MS patients who were off treatment for three months had significantly higher Population II than HD (*p* = 0.026) (Fig. 3C), as did MS patients who were on treatment (*p* = 0.04).

Treg Population II increased significantly in patients with inactive MS compared to HD (*p* = 0.012) but not with active MS (Fig. 3D).

### Changes in populations with age: comparison of HD and MS patients

Previous studies showed CD45RA^+^CD25^+^Foxp3^+^ naïve Treg decrease with age. Within the CD4^+^ gate there was a significant decrease in the Population I (R^2^ = 0.571, *p* < 0.001) with age in HD but not MS (data not shown).

**Figure 4:**
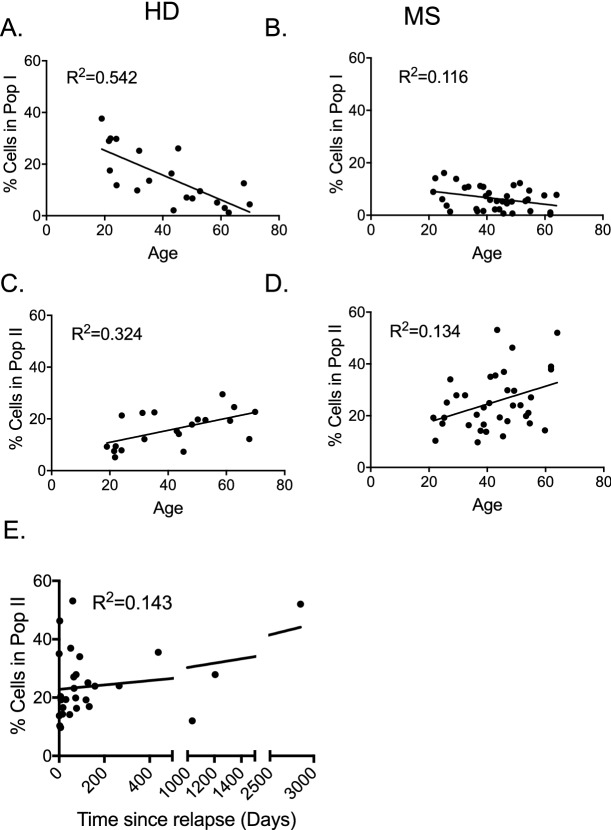
Regression analysis of Populations I and II in CD4^+^CD25^+^CD127^lo^Treg. The proportion of Treg in Populations I and II were analysed for correlation with age of HD and MS patients. (**A**,**B**) Linear regression analysis of Populations I with age in HD (n = 20) (A) and MS (n = 36) (B). In HD, Population I decreased with age (R^2^ = 0.542) but not in MS patients (R^2^ = 0.116). (**C**,**D**) Linear regression analysis of Populations II with age in HD (n = 20) (C) and MS (n = 36) (D). Population II increased with age for HD (R^2^ = 0.324) and MS (R^2^ = 0.134). (**E)** Linear regression analysis of Population II with time since last MS relapse showed correlation (R^2^ = 0.143) (*p* < 0.05).

Within the CD4^+^CD25^+^CD127^lo^Treg gate, Population I decreased with age in HD (R^2^ = 0.542, *p* < 0.001) and to a lesser degree in MS (R^2^ = 0.116, *p* = 0.042) (Fig. 4A,B). The decline with age was less apparent in MS patients (*p* = 0.042) compared to HD (*p* < 0.001).

Population II (CD45RA^−^Foxp3^hi^) within the CD4^+^ gate showed a significant increase with age in MS patients (R^2^ = 0.237, *p* = 0.003), but not in HD (R^2^ = 0.034,* p* = 0.436)(data not shown). Within the CD4^+^CD25^+^CD127^lo^Treg gate, Population II increased with age in both MS (R^2^ = 0.134, *p* = 0.028) and HD (R^2^ = 0.324, *p* = 0.009) (Fig. 4C,D).

Population III, in both CD4^+^ and CD4^+^CD25^+^CD127^lo^Treg gates had no significant relationship with age in HD or MS patients (data not shown).

### Changes in populations with disease duration in MS patients

Population I significantly decreased with MS duration within both, the CD4^+^ (R^2^ = 0.151, *p* = 0.026) and CD4^+^CD25^+^CD127^lo^Treg gates (R^2^ = 0.16, *p* = 0.021) (data not shown). No significant changes in Population II with disease duration were identified, however.

Linear regression analysis of Treg with time since last flare of MS showed no correlation for Population I (data not shown), but Population II increased with length of clinical remission (R^2^ = 0.143, *p* = 0.047) (Fig. 4E). Population III in the CD4^+^CD25^+^CD127^lo^Treg population increased with time since last clinical relapse (R^2^ = 0.151, *p* = 0.041).

### Chemokine receptor expression by the three Populations of Treg

**Figure 5:**
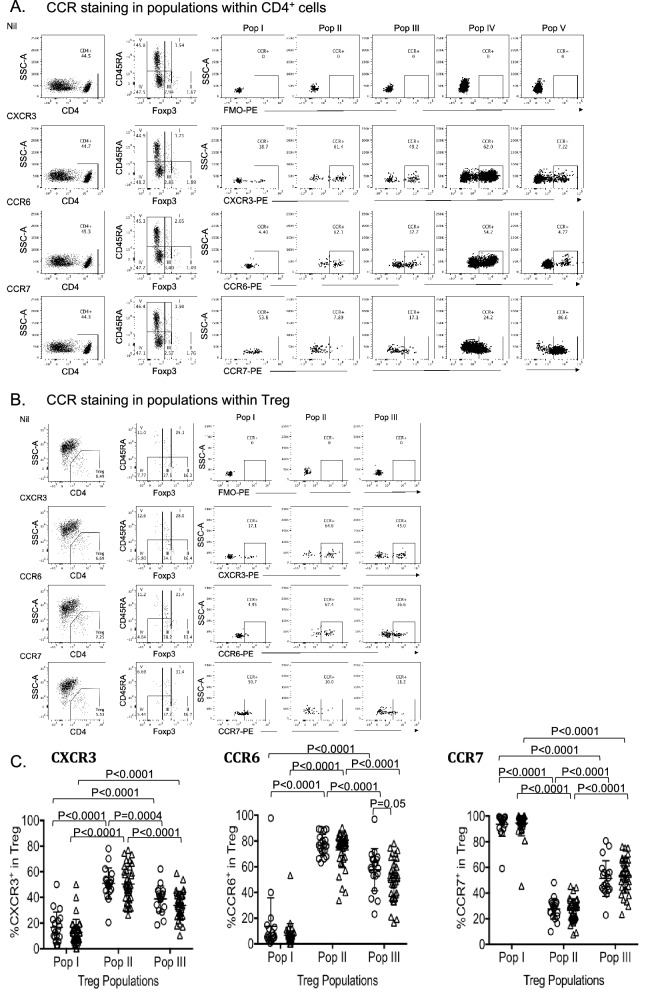
Chemokine receptor expression in Treg Populations I–III in HD and MS patients. (**A**) Representative FACS analysis for CXCR3, CCR6 and CCR7 in CD4^+^ Populations I-V. Expression of chemokine receptors (CCR) was examined in the five Populations of CD4^+^ cells by using FMO control for CCR monoclonal antibody fluorochrome (top row). (**B**) Representative FACS analysis for CXCR3, CCR6 and CCR7 in Populations I–III within CD4^+^CD25^+^CD127^lo^Treg. Chemokine receptors (CCR) expression was examined in the three Treg Populations using FMO control for CCR monoclonal antibody fluorochrome (top row). (**C**) Comparison of chemokine receptor expression in Treg Populations I–III in HD (Ο, n = 20) and MS patients (Δ, n = 31). The proportion of cells expressing CXCR3, CCR6 and CCR7 was examined within CD4^+^CD25^+^CD127^lo^Treg populations I-III that were identified based on Foxp3 vs. CD45RA expression as outlined in Fig. 2A. Values shown as the percentage of cells expressing respective CCR within each population. Both in HD (n = 19) and in MS patients (n = 35), expression of CXCR3 and CCR6 was higher in Treg Population II compared to Population I (*p* < 0.0001) or in Population III (*p* < 0.0001). Population III also had higher expression of CXCR3 and CCR6 compared to Population I (*p* < 0.0001). CCR7 expression in Treg Population II in both HD (n = 19) and MS (n = 33) was significantly lower compared to Treg Population I (*p* < 0.0001) or Population III (*p* < 0.0001). Population III also had significantly lower CCR7 expression compared to Treg Population I in both MS and HD. Approximately half of the MS patients have low CXCR3 and CCR6 expression in Population II. CCR6 expression was lower in Population III in MS compared to HD (*p* = 0.05).

Representative plots as Fig. 5A,B show chemokine receptor positive cells in Populations I-V of CD4^+^ cells and in Treg Population 1–III respectively. The gate for CCR^+^ cells is set based on chemokine receptor FMO control in respective subpopulations, top row in Fig. 5A,B.

CCR7 was expressed by 90% of Population I but only a small portion of Population II and III (Fig. 5C). This is consistent with CCR7^+^ naïve Treg recirculating from blood to lymphoid tissues. In Population I, lack of expression of CCR7 identifies TemRA Treg, which in MS were 5.55 + 9.74% (n=33), similar to 6.43 + 9.23% (n=19) in HD.

CCR7 was significantly lower in Population II (26.52±1.60%) compared to Population I (94.45±1.70%, *p* < 0.0001) and Population III (53.63±2.37%, *p* < 0.0001). Population III also had lower expression of CCR7 than Population I (*p* < 0.0001) (Fig. 5C).

Majority of CD4^+^ T cells in Population V express CCR7, which promotes their migration into secondary lymphoid tissues.Within Population V, there are CD4 TemRA cells that express CD45RA but not CCR7^47,61^. These are not naïve T cells. In this study MS patients had 12.25 + 23.3% of Population V that were CCR7^−^ compared to 5.16 + 8.46% in HD.

Activated Treg express chemokine receptors that promote their migration to sites of inflammation. CXCR3 promotes migration to sites of Th1 inflammation and CCR6 to sites of Th17 inflammation^53^. In both HD and MS patients, within CD4^+^CD25^+^CD127^lo^Treg gate, the highest expression of CXCR3 was in Population II (50.49±2.28%) compared to Population I (12.63±1.72%, *p* < 0.0001) and Population III (33.8±1.73%, *p* < 0.0001) (Fig. 5C). Population III had higher expression than Population I (*p* < 0.0001).

CCR6 was significantly higher in Population II (70.99±3.01%) compared to Population I (6.54 ± 1.48%, *p* < 0.0001), and Population III (47.93±2.94%, *p* < 0.0001). Population III had higher proportion of CCR6^+^ cells than Population I (*p* < 0.0001) (Fig. 5C).

The staining methods used in this study did not allow identification of CXCR3/CCR6 double positive cells. In our ongoing studies, we examined a group of MS patients with clinically active disease (n=10) to HD (n=10). In Population II, CXCR3^+^CCR6^+^ cells were 20.2 + 20.1% in MS compared to 13.1 + 7.9% in HD (NSD, *p* = 0.31). In Population III, CXCR3/CCR6 double positive cells were 6.2 + 4.8% in MS and 8.0 + 9.1% in HD. Thus, a small population of CXCR3^+^CCR6^+^ cells can be identified in HD and MS.

### CCR6 expression is decreased in Treg of MS patients

A significant decrease in the number of CCR6^+^ cells in MS patients was observed within Population III of CD4^+^CD25^+^CD127^lo^Treg compared to HD (*p* = 0.05) (Fig. 5C). Some MS patients had low expression of CCR6 in Population II. Reduced frequency of CCR6^+^ Treg did not associate with activity of MS, treatment or length of MS (data not shown). There were a few cases of low CCR6 expression in untreated, in any clinical groups. A larger cohort is required to resolve the meaning of this observation.

CXCR3 or CCR7 expression in subpopulations of CD4^+^ cells or CD4^+^CD25^+^CD127^lo^Treg did not show any significant difference between MS and HD (Fig. 5C).

## Discussion

This study identified trends in subpopulations of CD4^+^ cells that may have pathogenic significance in MS. As Treg function on ratio basis to effector CD4^+^ cells, we focused on the proportion of Treg subpopulations within whole CD4^+^ cells gate or within CD4^+^CD25^+^CD127^lo^Treg gate and not on the absolute number of Treg. CD45RA expression was used to distinguish resting from activated/memory cells. Subtypes of activated Treg were analysed by expression of Th17 and Th1 associated chemokine receptors. Both activated/memory Treg (Population II in CD4^+^CD25^+^CD127^lo^Treg) and activated effector T cells (Population IV in CD4^+^) were increased in MS. Both CD4^+^CD45RA^+^CD25^−^Foxp3^−^ cells (Population V in CD4^+^) and CD4^+^CD25^+^Foxp3^+^CD127^lo^Treg (Population I in Treg) were reduced in MS.

Many early studies in MS assumed CD4^+^CD25^+^Treg were a homogenous population of naïve Foxp3^+^Treg. In 29 studies we identified that examined CD4^+^CD25^+^Treg either without or with Foxp3/low CD127 expression in MS compared to healthy donors; 12 studies found no difference in MS compared to HD in frequency^5,6,9,24,25,27–30,32,34,37^ while nine found reduced Treg^7,10,11,14–17,19,20^, and three found reduced Treg during relapses^8,12,19^. Other studies reported reduced Treg in stable MS but an increase in Treg associated with relapses^7^ and progressive MS^20^. Three of 29 studies did not compare Treg frequency to HD, but studied function of Treg^23^ or monitored Treg in MS patients in response to therapy^33,35^. We also found no difference between CD4^+^CD25^+^Foxp3^+^CD127^lo^ T cells in MS and HD.

Our main findings were differences in subpopulations of Treg identified by expression of CD45RA or chemokine receptors. We found a shift from resting to activated/ memory Treg in MS patients. The most consistent finding is reduced numbers of resting CD4^+^CD25^+^CD45RA^+^Foxp3^+^Treg in MS relative to age-matched controls. Resting Treg decrease with age in HD, accompanied by an increase in activated/memory Treg^62^, likely due to natural exposure to various antigens throughout life activating resting Treg. The reduction in CD45RA^+^ cells in both Population I (resting Treg) and V (CD4^+^ effectors) may be due to activation of resting cells by the autoimmune response. It was not due to loss of TemRA effector or regulatory cells. We observed a decline in resting Treg with age in MS patients as well, as occurs in HD.

In 11 studies of CD45RA/RO expression by Treg in blood of MS patients^11,14,22,24,27,29,31,33–35,37^, two studies used CD45RA for longitudinal monitoring of Treg in MS but did not compare to HD^33,35^. Two others did not report resting Treg numbers^24,27^. Of the other seven studies, five showed lower numbers of CD45RA^+^ cells in MS compared to HD^11,14,29,34,37^. One found reduction in both CD45RA^+^Treg and CD45RA^−^Treg^22^ and the other no reduction in resting Treg^31^.

Resting Treg have been reported as lower than in HD in newly diagnosed MS in both children^14^ and in adults^29^. In adults, there is no change in total Treg, but reduced CD31^+^Treg and increased memory Treg in adults^29^. In paediatric MS, resting Treg and CD31^+^RTE Treg were reduced while memory Treg increased^14^. Our study confirmed reduced resting Treg in MS patients with no prior immune modifying therapy, as well as in established MS. Whether depletion of resting Treg is a contributor to induction of MS or a consequence of the auto immune responses, needs to be resolved. Depletion of resting Treg did not appear to be a consequence of therapy, however. A minority of Population I Treg did not express CCR7, and are effector memory Treg. Their proportion in Population I was not different in MS compared to HD, and these cells were thus also relatively depleted.

We found nine studies identifying memory/activated Treg in MS patients by expression of CD45RO or lack of CD45RA. Five reported an increase in activated/memory Treg^14,29,31,34,37^, two found no difference^24,27^, and two reported reduced memory Treg^10,22^ in MS patients. Only four of the nine studies in MS of CD45RA/RO expression by Treg, described three Treg population based on CD45RA and Foxp3/CD25 expression in MS or CIS^22,31,34,37^. Our findings are in agreement with Ciccocioppo et al 2019 who, like our study, found no difference in MS or HD in total CD4^+^CD25^+^CD127^lo^Treg, but higher activated Treg (Population II, CD45RA^−^CD25^hi^) and lower resting Treg (Population I, CD45RA^+^CD25^+^) in MS^37^. Jones et al^34^ showed an increase in Population III but not Population II in CIS patients. Sambucci et al^22^, showed a reduction in memory Treg but did not identify changes in Population II. Bjerg et al^31^ reported higher memory Treg associated with lower EDSS score.

In this study, activated/memory CD45RA^−^ Treg were increased, particularly in the CD25^hi^ and Foxp3^hi^ cells. Population II was greater in MS patients without clinical activity and increased the longer they were not clinically active. This is consistent with the increased activated Treg pool controlling MS, as reported^29,37^.

The CD45RA^+^ and CD45RO^+^ subsets of Treg, the naïve and memory Treg respectively, are affected differently in MS. In the acute phase of MS, suppressive function is impaired in both subsets, but activated/memory Treg recover in chronic MS^11^. This is accompanied by an increased activated/ memory Treg frequency in chronic patients, potentially explaining the observed recovery of Treg in secondary progressive MS^5,63^ and the positive correlation between MS duration and Treg frequency^11,15^. However, in chronic disease the number of resting Treg remains low. Higher proportions of activated/memory Treg are also found in CSF of MS patients compared to their peripheral blood^10,12,24^.

Previous studies on activated Treg in MS assayed other markers, but none found a consistent increase in Treg. CD39, a rate-limiting enzyme in ATP/ADP–AMP–adenosine pathway, produces adenosine, inhibits activated T effector cells and thereby limits immune inflammation. CD39 promotes a major pathway for Treg inhibition of autoimmunity. In eight studies of CD39 expression by Treg in MS, five reported reduced CD39^+^Treg^7,16,22,28,30^, one no difference^37^ and two increased numbers^32,36^. Most studies examined CD39 expression by the whole Treg population. Dalla Libera et al.^7^ showed CD4^+^CD25^+^Foxp3^+^ or CD4^+^CD25^+^CD39^+^ or CD4^+^CD39^+^Foxp3^+^ are reduced in stable RRMS but restored to normal in acute relapse in RRMS. Alvarez-Sanchez et al.^36^ showed increased CD39^+^ cells in CD4^+^CD25^+^CD127^lo^Foxp3^+^ in RRMS compared to HD. CD39^+^Treg have been found to be associated with relapsing-remitting MS and are increased in relapsing patients and is significantly correlated with EDSS score^36^. Patients have reduced CD39^+^Treg in a stable phase but in acute relapse had comparable CD39^+^Treg to HD^28^. Of the two studies examining CD39^+^ cell numbers in conjunction with CD45RA/RO, one reported reduced CD39^+^ cells in memory/activated Treg^22^. The other study found no difference in CD39 expression in MS and HD within total Treg, secreting Treg (Population III) or activated Treg (Population II)^37^. CD39^+^Treg were reduced in stable RRMS patients but their suppressive ability was not compromised^7^. Others reported that CD39^+^Treg have reduced capacity to suppress IL-17 production^30^. HD have marked variation ranging from 2% to 60% CD39^+^ cells within the CD4^+^CD25^hi^ Treg population^28^.

Another marker of activated Treg is expression of class II MHC^64,65^. In four studies in MS^27,33,35,37^, two monitored HLA-DR^+^ Treg in response to therapy and made no comparison to HD^33,35^ and one found no difference in HLA-DR^+^Treg^27^. Only one study reported an increase in HLA-DR^+^ Treg within total and activated/memory Treg in MS compared to HD^37^. There is one study showing increased PD-1 expression on Treg in MS compared to in HD^22^. Overall the published studies do not show an increase in these activated Treg in MS patients.

In MS patients, CCR6 expression was lower in Population III, and for a fraction of patients in Population II when gating was on CD4^+^CD25^+^CD127^lo^ cells, but not when gating was on all CD4^+^ cells. CCR6 is the chemokine receptor of Th17 cell responses. Th17 cells contribute to auto-inflammation in MS^48^, and infiltration of CCR6^+^CXCR3^+^ Th1 and Th17 effector memory cells into the CNS occurs in early disease activity in MS^66^. Given the role of CCR6 in chemotaxis^67^ with homeostatic accumulation of CCR6^+^Treg into the CNS following EAE induction in mice^68^, the decreased expression of CCR6 by CD4^+^CD25^+^CD45RA^−^ Treg may lead to the reduced migration of these Treg to inflammatory lesions.

Jones et al. found an increase in Th17-like Treg in CIS patients^34^. They defined Treg as CD4^+^Foxp3^+^CXCR5^−^ and hence their analysis would have excluded cells that may co-express both CXCR5 and CCR6. They also studied another marker of Th17 cells, CD161, in CIS^34^, These Th17-like Treg may control the Th17 effector response in CIS and prevent progression to MS. We found no differences in expression of CXCR3 the Th1 CCR. In a pilot study, we found a minority of cells in Population II and III express both CXCR3 and CCR6, but this was similar in HD and MS.

Our study identified several differences between MS patients and HD that may prove useful in monitoring MS. The proportion of activated/memory CD45RA^−^Foxp3^hi^Treg was increased in MS and appeared to be greatest in patients with longer clinical remissions. Increased activated/memory Treg may prove to be a marker of stable MS, with a reduced risk of relapse. CCR6 expression was also low in activated Treg and a relative deficiency of these cells may allow progression of MS.

This report is based on a limited number of patients and requires confirmation in a larger longitudinal study examining the subsets of activated/ memory Treg, identified in this study. The effects of therapy on the Treg populations and disease activity were not assessed.

However, this study including a large proportion of patients who never had immune modulating therapy, or who had no immune modulating therapy within three months prior sampling, confirmed that resting Treg are depleted in MS without immune modulating therapy, at a rate greater than natural attrition of these cells with age. Whether this makes patients more prone to develop MS or is a consequence of accelerated activation of Treg by the autoimmune response remains to be resolved.

Our findings identify an increased proportion of CD25^hi^Foxp3^hi^ Treg in many MS patients a finding supported by other smaller studies^31,37^. Similar changes were reported in sarcoidosis, a Th1 mediated disease, but not in SLE^43^. This suggests activated/memory Treg, which should include autoantigen specific Treg are generated to control immune inflammation in MS.

The decreased frequency of CCR6^+^ cells in Population III and low numbers of CCR6^+^ activated/memory Treg in Population II in some MS patients, suggested that failure to produce Th17-like Treg may contribute to lack of control of inflammation in MS.

The methodology will allow more detailed studies of Treg in MS especially with the highly activated Treg in Population II, which can be further divided into Th1 and Th17-like Treg, and other markers of activated Treg such as CD39, Class II MHC and PD1. Whether the increased proportion of activated/memory Treg leads to inhibition of immune inflammation and disease activity requires resolution. It may potentially guide therapy.

## Abbreviations

CIS: Clinically isolated syndrome
CNS: Central nervous system
CSF: Cerebrospinal fluid
FACS: Fluorescence-activated cell sorting
HD: Healthy donor
IFN-γ: Interferon-gamma
MOG: Myelin oligodendrocyte glycoprotein
MS: Multiple sclerosis
PBL: Peripheral blood lymphocytes
Treg: T-regulatory cell
